# Development law and growth model of dynamic pore water pressure of tailings under different consolidation conditions

**DOI:** 10.1371/journal.pone.0276887

**Published:** 2022-10-31

**Authors:** Changbo Du, Xinqi Jiang, Laigui Wang, Fu Yi, Ben Niu

**Affiliations:** 1 College of Civil Engineering, Liaoning Technical University, Fuxin, China; 2 School of Mechanics & Engineering, Liaoning Technical University, Fuxin, Liaoning, China; 3 College of Architecture and Transportation, Liaoning Technical University, Fuxin, China; Central Queensland University, AUSTRALIA

## Abstract

Tailings dams are in danger of liquefaction during earthquakes. The liquefaction process can be indirectly reflected by the evolution rule of the dynamic pore water pressure. To study the development law of dynamic pore water pressure of tailing sand under different consolidation conditions, the evolution equation of critical dynamic pore water pressure of tailings under isotropic and anisotropic consolidation conditions was derived based on the limit equilibrium theory. Moreover, the development law of dynamic pore water pressure was expounded theoretically. The dynamic triaxial tests of tailing silty sand and tailing silt under different dry densities, consolidation ratios, and confining pressures were performed. The dynamic pore water pressure ratio and vibration ratio curves of tailings under isotropic and anisotropic consolidation were analyzed, and a dynamic pore water pressure growth index model suitable for both isotropic and anisotropic consolidation was derived. The results showed that the critical dynamic pore water pressure was positively correlated with the confining pressure and average particle size of tailings under isotropic consolidation conditions. The tailings have a limit dynamic effective internal friction angle $\varphi_{d}^{c}$ under the anisotropic consolidation condition. The evolution law of critical dynamic pore water pressure can be judged according to the dynamic effective internal friction angle of tailing sand *φ*_d_ and $\varphi_{d}^{c}$ values. The consolidation ratio significantly affects the dynamic pore pressure growth curve while confining pressure and dry density do not. For different tailing materials, the dynamic pore water pressure ratio is positively correlated with tailing particles. The dynamic pore water pressure growth process of tailing silty sand and tailing silt can be divided into two stages: rapid and stable growths. The development law of two types of tailings can be described by the dynamic pore water pressure growth index model. The research results can provide a theoretical basis for the seismic design of tailings dams in practical engineering.

## 1 Introduction

An effective stress method is key to analyzing the stability of saturated soil during dynamic load. The development of dynamic pore water pressure of soil significantly impacts the deformation and strength of its saturated material [1–3]. Therefore, the study of the occurrence, development, and dissipation of dynamic pore water pressure has gained substantial attention in the field of geotechnical engineering. The dynamic pore water pressure model is an important index indirectly reflecting the liquefaction of soil and reflecting the vibration liquefaction process of saturated soil [4]. Dynamic pore water pressure models include the stress [5–7], strain [8, 9], endochronic [10, 11], and energy models [12–15], according to the characteristics of the relationship between the model and dynamic pore water pressure. The stress model of dynamic pore water pressure, a commonly used model, relates the dynamic pore water pressure with the applied dynamic load. The stress model explores the relationship between dynamic pore water pressure and vibration ratios [16]. The models can be divided according to two conditions: isotropic (consolidation ratio *K*_c_ = *σ*_1_/*σ*_3_ = 1) and anisotropic (consolidation ratio *K*_c_ = *σ*_1_/*σ*_3_>1) consolidations. Seed et al. [5] conducted undrained dynamic triaxial tests on saturated sand under isotropic consolidation. They proposed a dynamic pore water pressure stress model of general saturated sand under isotropic consolidation used to predict and evaluate the liquefaction of saturated sand. However, this model is only applicable to isotropic consolidation but not to anisotropic consolidation. Several studies have been conducted for biased consolidation, and correction models have been proposed in combination with the Seed model [17–21].

Most mature dynamic pore water pressure stress models are designed for saturated natural sandy soil. Tailing sand and natural sandy soil differ in terms of physical and mechanical properties, making classical dynamic pore water pressure stress models unsuitable to be directly applied to tailing sand. Therefore, the existing dynamic pore water pressure models should be modified. Relevant studies have been conducted on the dynamic pore water pressure model of tailings [22–27]. Zhang et al. [28] explored the dynamic deformation characteristics of tailings through dynamic triaxial and resonance column tests and proposed a simple dynamic pore water pressure correction model based on the Seed dynamic pore water pressure model. Liu et al. [29] proposed a new dynamic pore water pressure model based on numerous experimental studies combined with the existing dynamic pore water pressure models. Zhang et al. [30] studied the dynamic pore water pressure evolution law of iron tailings under cyclic load through dynamic triaxial tests under different consolidation ratios and established the dynamic pore water pressure development model of tailings under isotropic and anisotropic consolidation conditions. Wu et al. [31] designed and built a sand well drainage system in the red mud tailings pond. They established a single well drainage dynamic pore water pressure calculation model and derived the dynamic pore water pressure differential equation.

Tailings are artificially discharged granular materials, different from natural sandy soil, and are usually saturated owing to stacking and other factors. In addition, they are prone to liquefaction damage under dynamic loads, such as earthquakes [32]. Therefore, the prediction model of dynamic pore water pressure growth is essential for studying the dynamic stability analysis of tailings. Considering the “code for design of tailings facilities” (GB50863-2013), tailings can be divided into three categories according to the particle size and plasticity index: sandy tailings (e.g., tailing silty sand), silty tailings (e.g. tailing silt), and cohesive tailings (e.g., tailing silt clay). With the progress of beneficiation technology, the particle size of tailings is becoming smaller. Tailings dams are progressively being built with tailing silty sand and tailing silt. Compared with tailings silty sand, tailings silt has a finer particle size (less than 0.075 mm tailings content more than 50%), worse strength, and lacks viscosity (plasticity index less than 10), making it prone to liquefaction [33]. Studies on the dynamic characteristics of tailing sand did not consider the development law of dynamic pore water pressure under different consolidation conditions, and the influence of various factors on the development law of dynamic pore water pressure is unclear [34]. In the actual tailings pond structure, the dynamic damage process and dynamic pore water pressure evolution law of tailing sand under different conditions also differ [35]. In this study, we express the critical dynamic pore water pressure of tailing sand under isotropic and anisotropic consolidation conditions based on the limit equilibrium theory. First, the dynamic pore water pressure evolution law was explained theoretically. Thereafter, through the undrained consolidation dynamic triaxial tests of tailing silty sand and tailing silt under different dry densities, consolidation ratios, and confining pressures, the dynamic pore water pressure ratio and vibration ratio curves of tailing sand under isotropic and anisotropic consolidation were analyzed. Finally, a dynamic pore water pressure growth index model suitable for both isotropic and anisotropic consolidation conditions was established.

## 2 Theoretical analysis of influencing factors of dynamic pore water pressure evolution of tailings

The development law of dynamic pore water pressure of tailings materials is affected by many factors. The limit equilibrium standard was adopted to analyze the dynamic pore water pressure (i.e., critical dynamic pore water pressure *u*_cr_) of tailings in equilibrium (initial failure stage).

**Fig 1:**
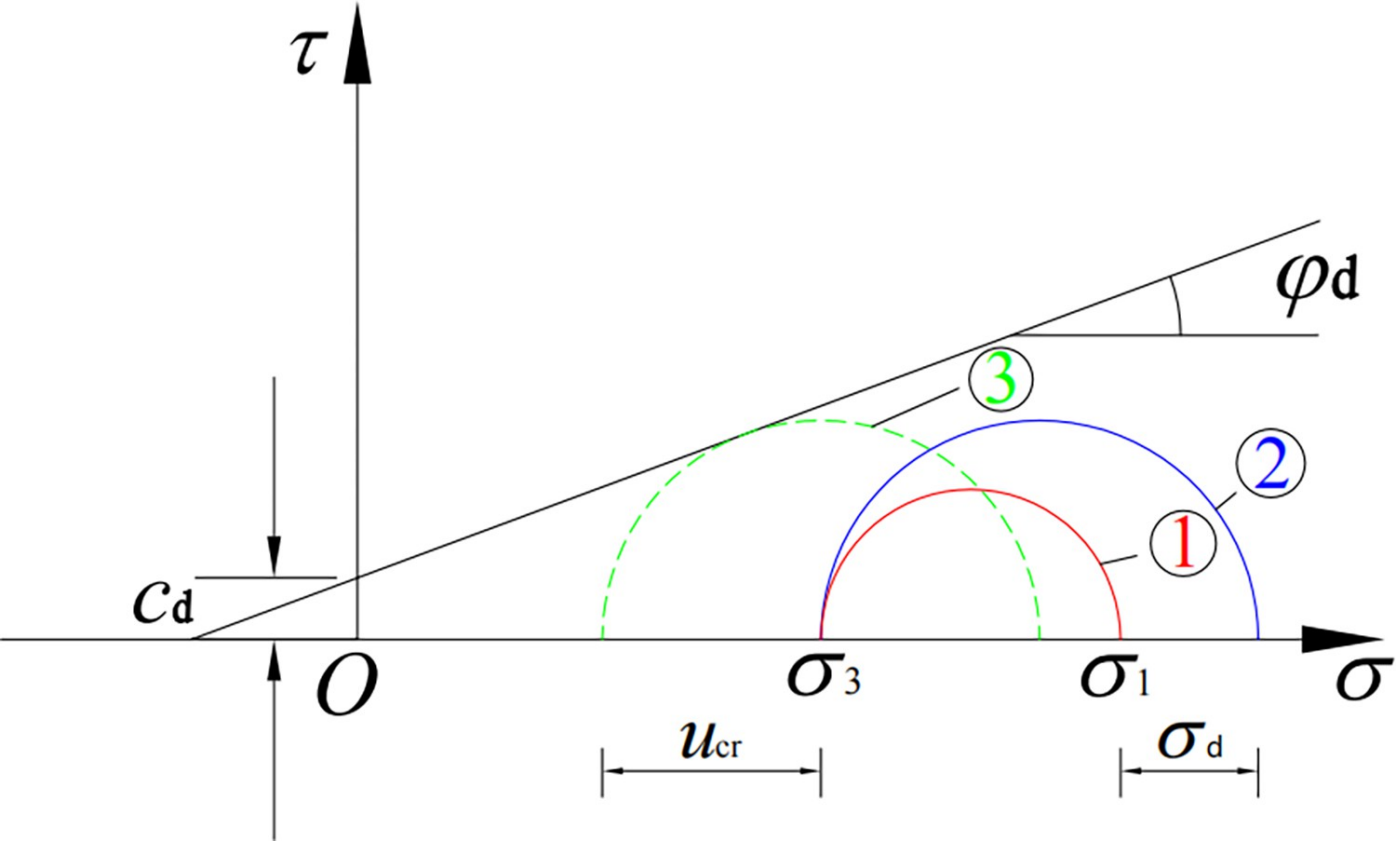
Critical dynamic pore water pressure under limit equilibrium.

We assumed that the static limit equilibrium condition of tailings material was applicable to the dynamic test, and the Mohr–Coulomb failure envelope of dynamic load and static load was the same; that is, the dynamic effective internal friction angle of the sample was equal to the static effective internal friction angle [30]. In Fig 1, the stress circle ① represents the stress state of the sample before vibration, whereas ② represents the largest stress circle during the application of dynamic load; that is, a stress circle whose dynamic stress is equal to its amplitude *σ*_d_ instantaneously. During the application of dynamic load, the dynamic pore water pressure in the sample continued to develop. When the dynamic pore water pressure was expressed by effective stress, the stress circle ② continued to move to the failure envelope (to the left). When the dynamic pore water pressure reached the critical value *u*_cr_, the stress circle was tangential to the failure envelope. Consequently, according to the limit equilibrium conditions, the tailings material was considered to reach the failure state.

According to the geometric conditions in Fig 1, the critical dynamic pore water pressure in limit equilibrium can be deduced as follows:

$$u_{\mathrm{cr}}=\frac{\sigma_{1}+\sigma_{3}}{2}+\frac{c_{d}}{\tan\varphi_{d}}-\frac{\sigma_{1}-\sigma_{3}+\sigma_{d}(1-\sin\varphi_{d})}{2\sin\varphi_{d}},$$ (1)

where *c*_d_ and *φ*_d_ are the static and dynamic effective cohesion and effective internal friction angle of the sample, respectively, and *σ*_d_ is the amplitude of dynamic stress.

According to Eq (1), the critical dynamic pore water pressure *u*_cr_ is related to the dynamic effective shear strength index of tailings (*c*_d_, *φ*_d_), axial pressure *σ*_1_, confining pressure *σ*_3_, and dynamic stress amplitude *σ*_d_ during the test. When analyzing whether the critical dynamic pore water pressure is affected by a particular factor, we assume that other influencing factors are fixed.

### 2.1 Isotropic consolidation

When σ_1_ = σ_3_, the critical dynamic pore water pressure under isotropic consolidation can be derived from Eq (1):

$$u_{\mathrm{cr}}=\sigma_{3}+\frac{c_{d}}{\tan\varphi_{d}}+\frac{\sigma_{d}(\sin\varphi_{d}-1)}{2\sin\varphi_{d}}=\sigma_{3}+\frac{c_{d}}{\tan\varphi_{d}}+k_{\varphi }\sigma_{d},$$ (2)

where $k_{\varphi }=(\sin\varphi_{d}-1)/2\sin\varphi_{d}<0$ is the proportional coefficient related to the material.

The following were derived after further analysis of Eq (2):

1. In Eq (2), when *σ*_d_ = 0, $u_{\mathrm{cr}}\tan\varphi_{d}=c_{d}+\sigma_{3}\tan\varphi_{d}$; that is, under static conditions, the tailings can bear the critical dynamic pore water pressure slightly longer than the confining pressure owing to cohesion.
2. For a single tailings material, the proportion coefficient *k*_*φ*_ remains consistent. Eq (2) shows that when the dynamic stress amplitude is constant, the critical dynamic pore water pressure is positively correlated with the confining pressure. The confining pressure increases with an increase in critical dynamic pore water pressure, enhancing the anti-liquefaction ability of tailings. When the confining pressure is constant, the critical dynamic pore water pressure is negatively correlated with the dynamic stress amplitude. The dynamic stress amplitude increases with a decrease in critical dynamic pore water pressure, resulting in the tailings material being easily destroyed.
3. For different tailings materials, under the same confining pressure and dynamic stress amplitude, the average particle size of tailings is positively correlated with the dynamic effective internal friction angle *φ*_d_, proportional coefficient *k*_*φ*_, critical dynamic pore water pressure, and anti-liquefaction ability of tailings materials.

### 2.2 Anisotropic consolidation

*σ*_1_ = *K*_c_*σ*_3_ is substituted into Eq (1) to obtain the critical dynamic pore water pressure under the anisotropic consolidation (*K*_c_ > 1.0) condition:

$$u_{\mathrm{cr}}=(\frac{1+\sin\varphi_{d}}{2\sin\varphi_{d}}-\frac{1-\sin\varphi_{d}}{2\sin\varphi_{d}}K_{c})\sigma_{3}+\frac{c_{d}}{\tan\varphi_{d}}+\frac{\sigma_{d}(\sin\varphi_{d}-1)}{2\sin\varphi_{d}}=\lambda \sigma_{3}+\frac{c_{d}}{\tan\varphi_{d}}+k_{\varphi }\sigma_{d}$$ (3)

In the Equatio, λ is the critical dynamic pore water pressure ratio, and $\lambda =\frac{1+\sin\varphi_{d}}{2\sin\varphi_{d}}-\frac{1-\sin\varphi_{d}}{2\sin\varphi_{d}}K_{c}=\frac{1+\sin\varphi_{d}}{2\sin\varphi_{d}}+k_{\varphi }K_{c}$ is the ratio of critical dynamic pore water pressure to confining pressure at the beginning of material failure.

**Fig 2:**
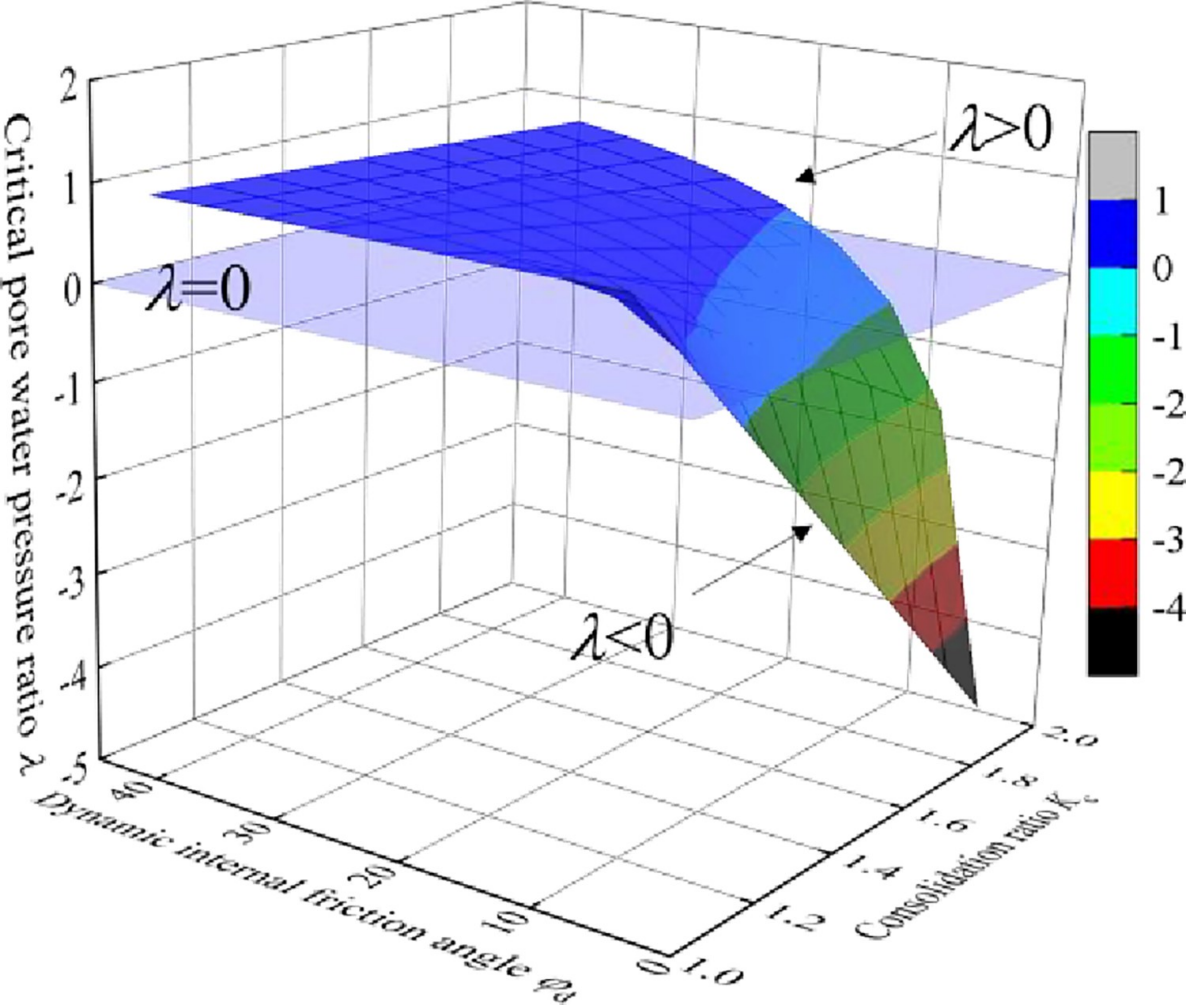
Change rule of critical dynamic pore water pressure ratio (λ).

**Fig 3:**
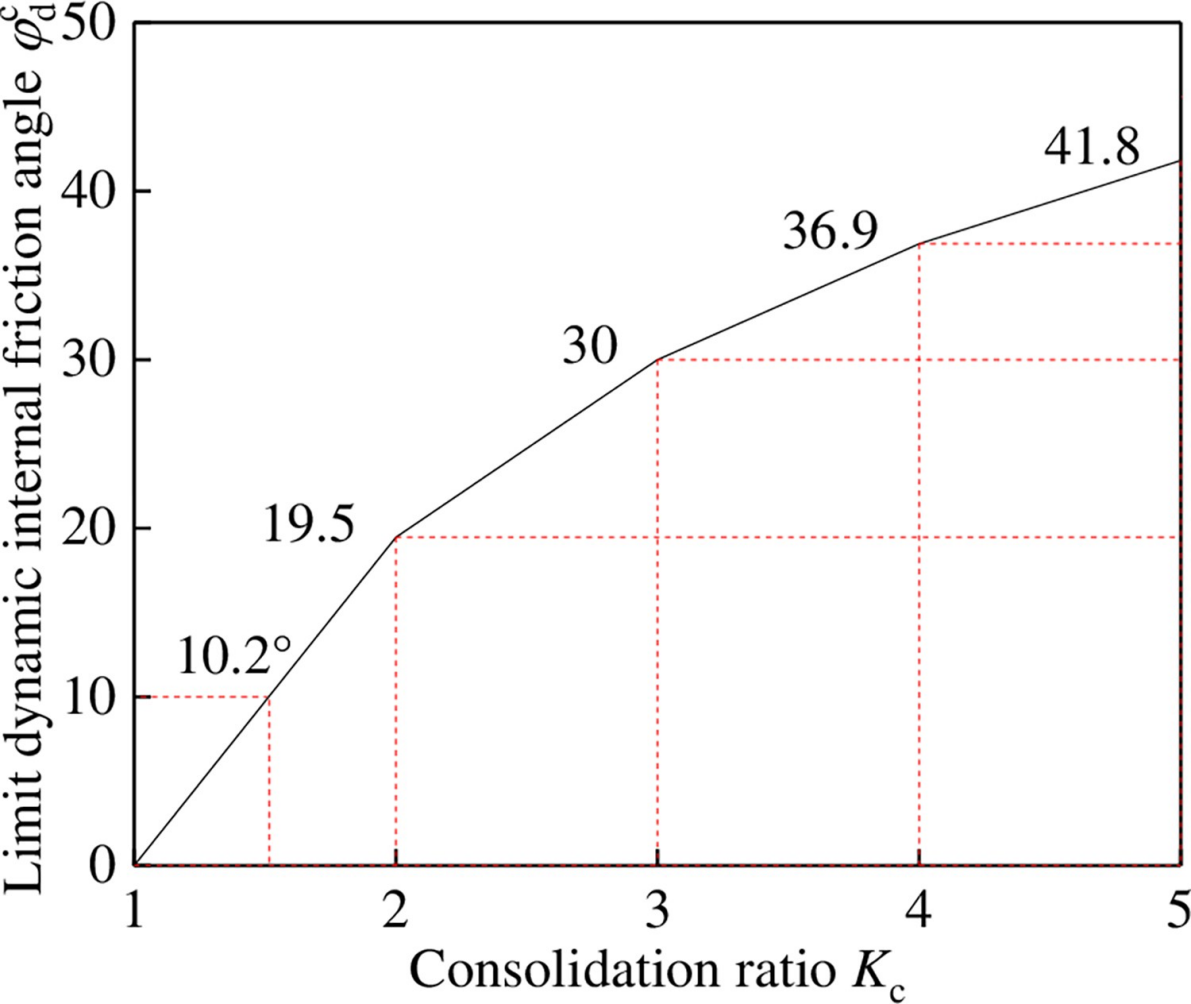
Limit dynamic internal friction angle $\varphi_{d}^{c}$ (λ = 0).

As shown in Fig 2, the critical dynamic pore pressure ratio λ varies with the effective internal friction angle *φ*_d_ and consolidation ratio *K*_c_. As *φ*_d_ decreases and *K*_c_ increases, λ gradually decreases. When λ = 0, $\varphi_{d}=\varphi_{d}^{c}=\arcsin\frac{K_{c}-1}{K_{c}+1}$, where $\varphi_{d}^{c}$ is the limit dynamic internal friction angle. Fig 3 shows the relationship between $\varphi_{d}^{c}$ and the consolidation ratio *K*_c_. $\varphi_{d}^{c}$ increases with an increase in *K*_c_. When *K*_c_ = 1.5, $\varphi_{d}^{c}=10.2{}^{\circ}$. When *K*_c_ = 2, $\varphi_{d}^{c}=19.5{}^{\circ}$.

Eq (3) is further analyzed as follows:

1. When $\varphi_{d}=\varphi_{d}^{c}$, λ = 0, and $u_{\mathrm{cr}}=\frac{c_{d}}{\tan\varphi_{d}}+k_{\varphi }\frac{\sigma_{d}}{2}$, the critical dynamic pore water pressure is independent of the confining pressure. For a single tailings material, the proportion coefficient *k*_*φ*_ remains constant. Eq (3) shows that the critical dynamic pore water pressure is negatively correlated with the dynamic stress amplitude.
2. When $\varphi_{d}>\varphi_{d}^{c}$ and λ > 0, the evolution law of critical dynamic pore water pressure under anisotropic consolidation condition is the same as that under isotropic consolidation condition: for a single tailings material, the critical dynamic pore water pressure is positively correlated with the confining pressure and negatively correlated with the dynamic stress amplitude. For different tailings materials, the critical dynamic pore water pressure is positively correlated with the average particle size of tailings.
3. When $\varphi_{d}<\varphi_{d}^{c}$ and λ < 0, for a single tailings material, the critical dynamic pore water pressure is negatively correlated with the confining pressure and dynamic stress amplitude; for different tailings materials, the critical dynamic pore water pressure is positively correlated with the average tailing particle size.

Therefore, physical significance of limit dynamic internal friction angle $\varphi_{d}^{c}$ can be described as the minimum dynamic effective internal friction angle when the critical dynamic pore water pressure is positively correlated with the confining pressure under the anisotropic consolidation condition. When the tailings material is consolidated under the anisotropic condition, the evolution of critical dynamic pore water pressure can be predicted according to the dynamic effective internal friction angle *φ*_d_ and limit dynamic effective internal friction angle $\varphi_{d}^{c}$ under different consolidation ratios.

## 3 Dynamic pore water pressure triaxial test analysis

### 3.1 Test material

**Fig 4:**
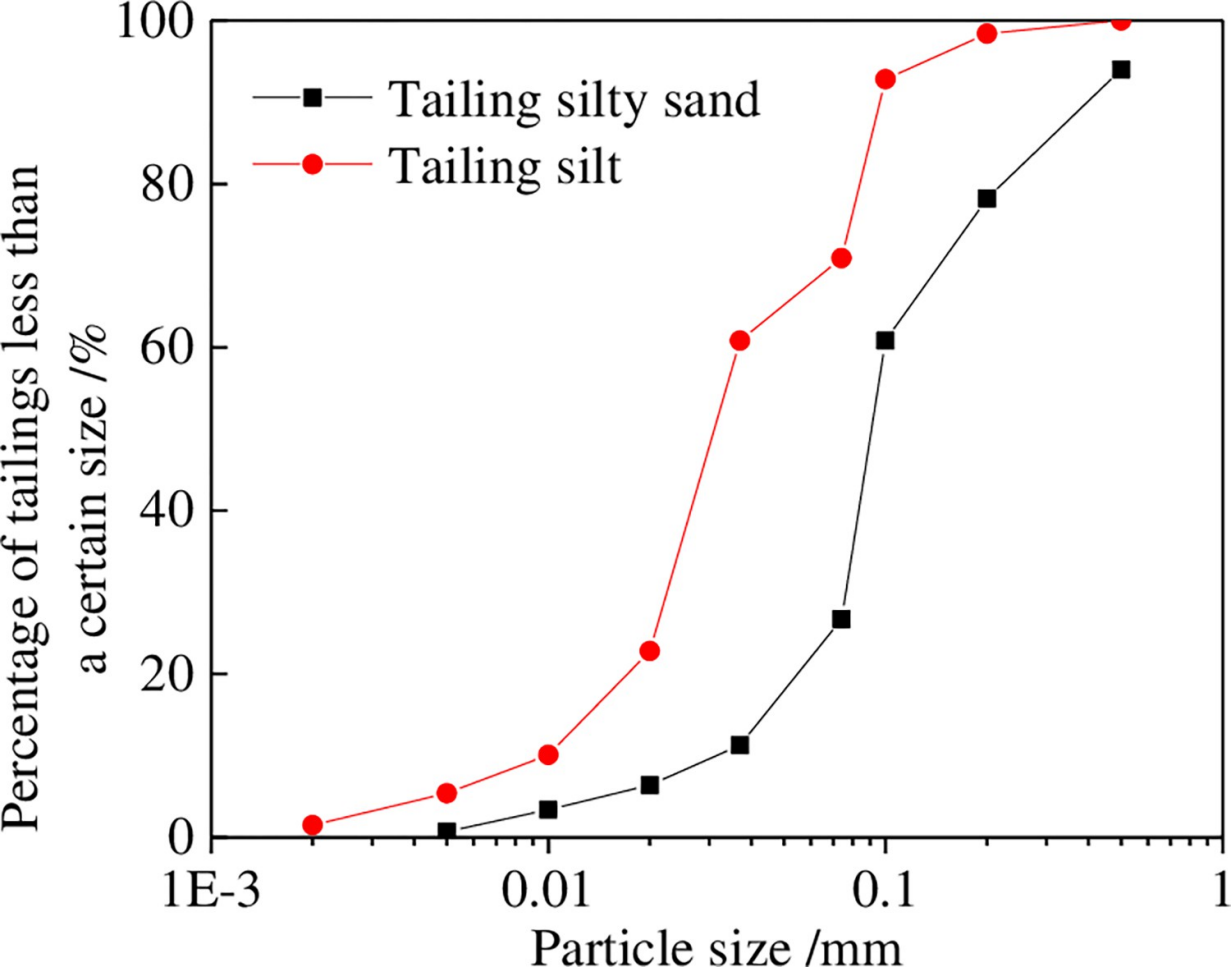
Particle grading curve of tailing sands.

According to the Standard Specification for Classification of Soil for Construction (unified standard soil classification system) of the American Society for Testing and Materials (ASTM D 2487), tailings sand can be classified into fine-grained soil, coarse-grained soil, and high organic soil, according to particle size, liquid limit, and plasticity index. The tailing silty sand and tailing silt used in the test were acquired from Yutiankeng tailings reservoir of Au-Cu mine in Zijin mining. Fig 4 shows the particle grading curve. The particle size distribution range of tailing silty sand and tailing silt were relatively the same, but the contents differed. The particles of tailing silty sand were mainly distributed in the range of 0.075–0.5 mm. Particles in this size range accounted for approximately 67.3% of the heavy mass, whereas particles less than 0.075 mm in size accounted for 26.7%. For tailing silt, particles in the 0.075–0.5 mm size range accounted for approximately 29.1% of the total mass, whereas particles less than 0.075 mm in size accounted for 60.8%. This indicated that there were more fine particles in the tailing silt than in tailing silty sand. Table 1 shows the results of the particle size analysis. The nonuniformity coefficient *C*_u_ of both tailings was less than 5, and the curvature coefficient *C*_C_ was greater than 1. This indicates that the tailing silty sand and tailing silt are homogeneous soils with poor grading and filling effect between coarse and fine particles. This leads to low density, high permeability, and poor seismic performance. After the tailing silty sand used in the test was sieved using the standard sieve specified by the ASTM, the particle content of the No. 200 sieve reached 26.7%. Moreover, its non-uniformity coefficient Cu was less than 5, and the tailings sand was silty sand (SM). After the tailing silt used in the test was sieved, the particle content of the No. 200 sieve reached 60.8%, the water content was less than 50%, and the plasticity index was less than 4. The end silt soil belonged to silt (ML).

**Table 1:** Test results of particle size analysis.

Tailings type	*d*_10_	*d*_30_	*d*_60_	*C*_u_ = *d*_60_/*d*_10_	$C_{c}=d_{30}^{2}/(d_{10}\times d_{60})$
Tailing silty sand	0.032	0.076	0.1	3.1	1.8
Tailing silt	0.01	0.023	0.037	3.7	1.4

### 3.2 Test instrument and sample preparation

**Fig 5:**
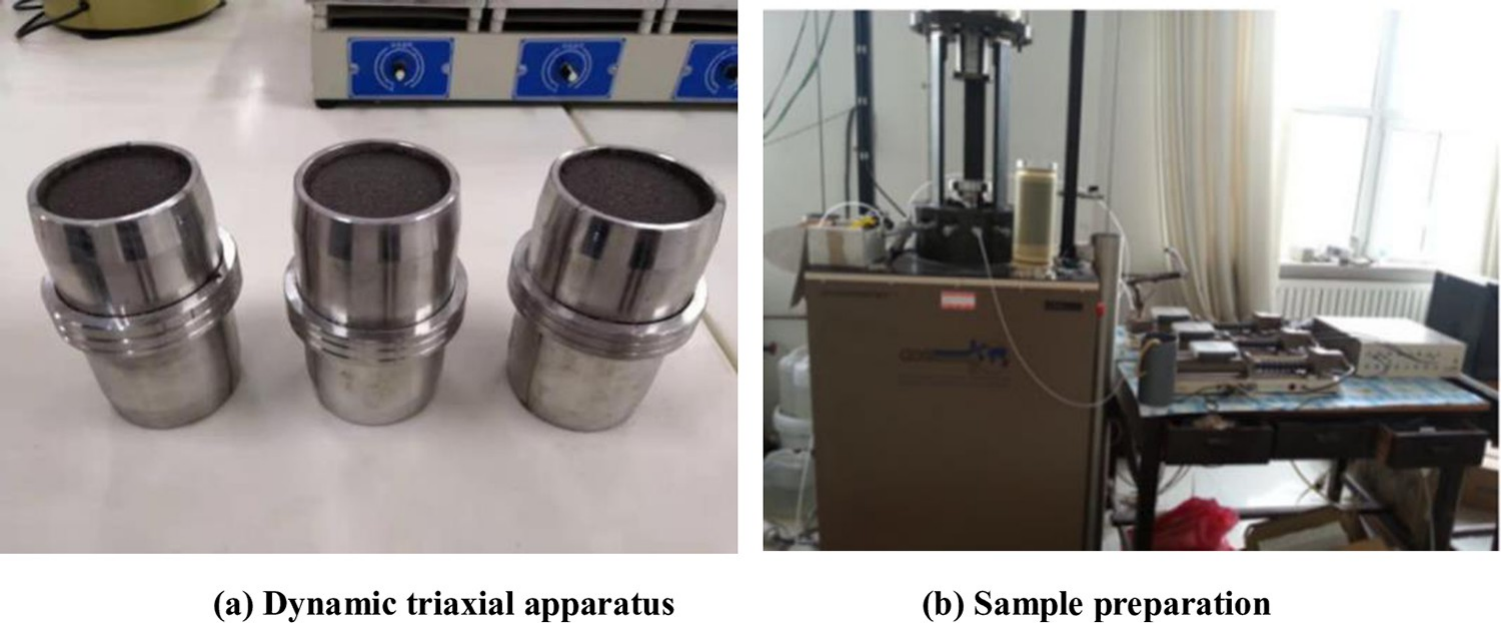
GDS SS-HCA dynamic triaxial test system. (a) Dynamic triaxial apparatus, (b) Sample preparation.

**Fig 6:**
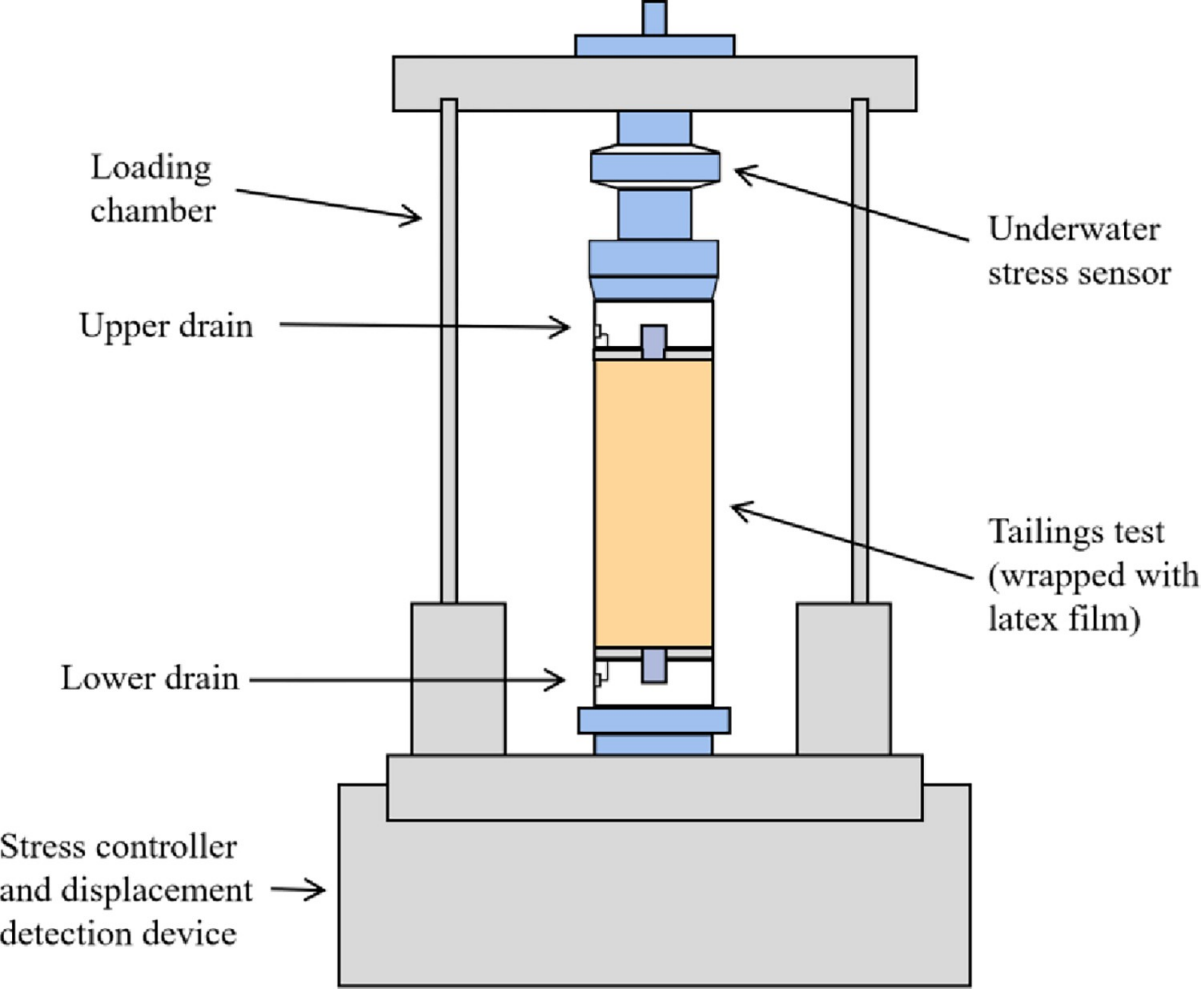
Section of dynamic triaxial testing machine.

A GDS SS-HCA dynamic triaxial test system was used in this test, including a confining pressure controller, back pressure controller, test stand, cavity, acquisition equipment, and computer. Figs 5 and 6 show the triaxial test device and its plane view, respectively. The test process adopts the strain control and unilateral dynamic loading method. The loading waveform was a sine wave, and the frequency was 1 Hz. The tailing test piece was prepared according to the sample preparation method in the standard for soil test methods (GB / T50123-1999) [36]. After site investigation and sampling, the reconstituted samples were prepared by wet compaction. The test piece was a standard cylinder with a 39.1 mm diameter and 80 mm height. Consolidation stability standard: the change in consolidated drainage volume should be less than 0.1 cm^3^ in 1 h. Failure standard: when the consolidation pressure (*K*_c_ = 1.0) is equal, the full amplitude strain is 5%, and when the consolidation pressure is unequal (*K*_c_ > 1.0), the comprehensive strain is 5%.

The sample was prepared by placing the tailing sand into a moisturizing tank, soaking it for 24 h, and then measuring its moisture content. According to its moisture content and the dry density required by the sample, the tailings sand was placed into the sampler in five layers and compacted to form. To avoid delamination between layers, the contact surface of each layer was roughened. The last layer was compacted, and the sample was removed and weighed. The parallel error of each sample was less than 0.02 g/cm^3^. A Φ 39.1 mm × 80 mm cylinder sample with a specified moisture content was prepared. The compacted sample was then placed in the saturator and into a vacuum pumping equipment for pumping, with a pressure close to atmospheric pressure. Subsequently, pumping was performed for 2 h, followed by slow injection of distilled water. The sample was left for more than four days and nights after the pumping was saturated. The soil sample saturation reached more than 0.95. Finally, the sample was consolidated according to the required stress state.

### 3.3 Test method and process

The Rules of Geotechnical Testing (SL237-1999) used by the Ministry of Water Resources of the People’s Republic of China was the standard of this test [36]. The specific operation steps during the test were performed in accordance with the "Geotechnical Test Regulations". The sample was consolidated under the conditions with the confining pressure *σ*_3_ equal to 100 kPa, 150 kPa, and 200 kPa. Thereafter, the dynamic stress of the sample was increased stepwise under undrained conditions for vibration test. The dynamic stress and strain under each level of dynamic load were recorded, and each group of dynamic load was five to six levels. Each group of tests adopted the method of applying dynamic loads by classification on one sample. To eliminate the influence of the dynamic pore water pressure generated by the previous level of dynamic load on the next level, the drain valve was quickly switched on and off after each level of dynamic load, eliminating the dynamic pore water pressure increment. Each level of dynamic load vibrated 10 times.

The test process of the specific dynamic test is as follows:

1. **Sample saturation:** The water head saturation and back pressure saturation methods are used to achieve a sample saturation of 95% and then the drainage consolidation is carried out.
2. **Sample consolidation:** The confining pressure is kept unchanged and the back pressure is adjusted. The difference between confining pressure and back pressure is set as the test confining pressure. The consolidation time is 12 h. The wave velocity of the sample is tested after the consolidation is completed.
3. **Dynamic load test:** In the dynamic strength test, the dynamic stress value is set in the controller according to different confining pressures. The dynamic stress loading frequency is 1 Hz, and the loading waveform is a sine wave. When the excess pore water pressure rises to the test confining pressure, the loading is stopped, and the sample is considered to be damaged.
4. **End test:** The confining and back pressure controllers are adjusted to reduce the internal and external pressures of the test piece to 0 kPa. Subsequently, the test piece is disassembled, and the equipment is prepared for the next test.

### 3.4 Test scheme

The dynamic test was performed with tailing silty sand and tailing silt under different stress conditions. The consolidation stress ratio *K*_c_ were set to 1.0, 1.5, and 2.0, and the confining pressures corresponding to consolidation stress ratios were 100 kPa, 150 kPa, and 200 kPa, respectively. Three to four different dynamic stresses were selected for each stress state. The saturated sample was consolidated under a specific stress state. Thereafter, a particular dynamic stress was applied on it under undrained conditions. Table 2 shows the specific test plan.

**Table 2:** Tailings dynamic test scheme.

Tailing	Test scheme		*K*_c_	*ρ*_d_/(g/cm^3^)	*σ*_3_/kPa
Tailing silty sand	Isotropic consolidation	a1	1	1.58	100,150,200
		b1	1	1.68	
		c1	1	1.79	
	Anisotropic consolidation	d1	1.5	1.58	
		e1	1.5	1.68	
		f1	1.5	1.79	
		g1	2	1.58	
		h1	2	1.68	
		i1	2	1.79	
Tailing silt	Isotropic consolidation	a2	1	1.51	100,150,200
		b2	1	1.65	
		c2	1	1.77	
	Anisotropic consolidation	d2	1.5	1.51	
		e2	1.5	1.65	
		f2	1.5	1.77	
		g2	2	1.51	
		h2	2	1.65	
		i2	2	1.77	

### 3.5 Dynamic strength index

The dynamic strength of soil is the dynamic stress required for soil to generate a specified strain under the action of a number of stress cycles. The Mohr–Coulomb shear strength theory can be applied to vibration. On the relationship curve between dynamic shear stress and failure vibration times of the same consolidation ratio, the dynamic shear stress corresponding to 10, 20, and 30 failure vibration times under three different confining pressures is intercepted *τ*_d_. The dynamic shear stress *τ*_d_ is the ordinate, and the principal stress *σ* is the abscissa. The envelope of the total stress–shear strength was plotted with (*σ*_1_+*σ*_3_)/2 as the center and (*σ*_1_−*σ*_3_)/2 as the radius. The dynamic effective cohesion *c*_d_ and dynamic effective internal friction angle *φ*_d_ under different failure vibration were obtained.


$$\tau_{d}=\sigma_{d}\cdot \tan\varphi_{d}+c_{d}$$ (4)

Table 3 shows the dynamic strength indexes of tailing silty sand under different conditions. The particle size of tailing silty sand was larger than that of tailing silt, a non-viscous bulk material; thus, the dynamic cohesion of tailing silty sand was 0, and the dynamic cohesion of tailing silt was approximately 1.7 kPa. The dynamic intensity index *φ*_d_ decreased with an increase in the vibration time. When the vibration times were certain, *φ*_d_ increased with an increase in dry density and consolidation ratio. The *φ*_d_ increased significantly with an increase in the consolidation ratio; however, it varied slightly with an increase in the vibration frequency and dry density.

**Table 3:** Dynamic strength indexes of tailings under different conditions.

Tailing	*K*_c_	*ρ*_d_/(g/cm^3^)	10 cycles		20 cycles		30 cycles		Average value	
			*c*_d_/ kPa	*φ*_d_/ kPa	*c*_d_/ kPa	*φ*_d_/ kPa	*c*_d_/ kPa	*φ*_d_/ kPa	*c*_d_/ kPa	*φ*_d_/ kPa
Tailing silty sand	1	1.58	0	9.3	0	9.2	0	9.1	0	9.7
		1.68	0	10	0	9.8	0	9.6		
		1.79	0	10.4	0	10.2	0	10		
	1.5	1.58	0	19.2	0	19	0	18.8	0	20.3
		1.68	0	20.3	0	19.7	0	19.4		
		1.79	0	22.5	0	22.1	0	21.7		
	2	1.58	0	25.4	0	25.1	0	24.8	0	26.6
		1.68	0	26.6	0	26.3	0	26		
		1.79	0	28.8	0	28.4	0	28		
Tailing silt	1	1.51	1	8.9	1	8.7	1	8.5	1.7	9.0
		1.65	2	9	2	8.9	2	8.8		
		1.77	2	9.4	2	9.3	2	9.2		
	1.5	1.51	1	17.2	1	17	1	16.8	1.7	17.6
		1.65	2	17.8	2	17.6	2	17.4		
		1.77	2	18.4	2	18.2	2	18		
	2	1.51	1	23.5	1	23.3	1	23	1.7	25.1
		1.65	2	25.5	2	25.2	2	25		
		1.77	2	27	2	26.7	2	26.4		

## 4 Development law of dynamic pore water pressure of tailing

### 4.1 Development law of dynamic pore water pressure

**Fig 7:**
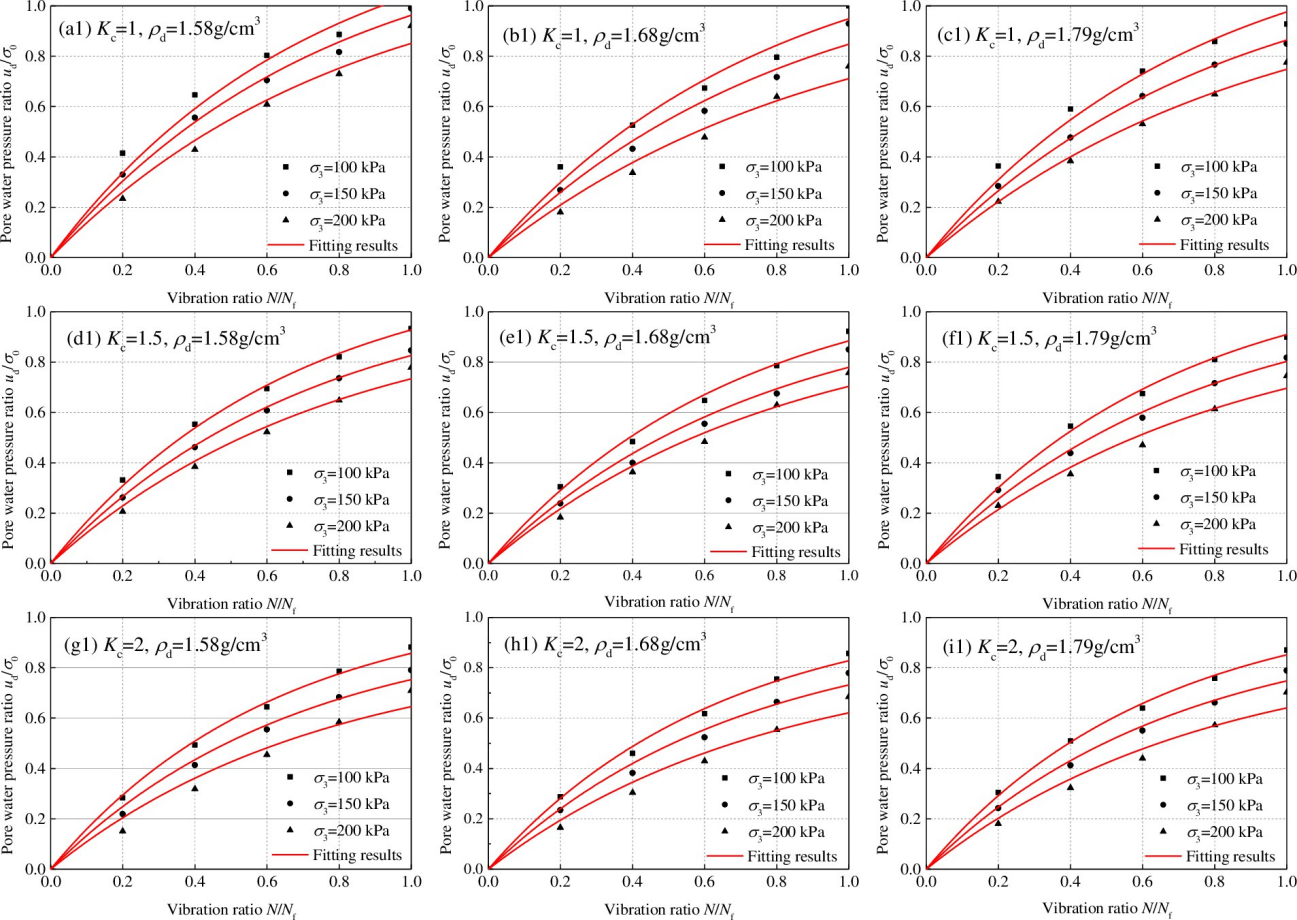
Relationship between dynamic pore water pressure ratio *u*_d_/*σ*_0_ and vibration ratio *N*/*N*_f_ of tailing silty sand.

**Fig 8:**
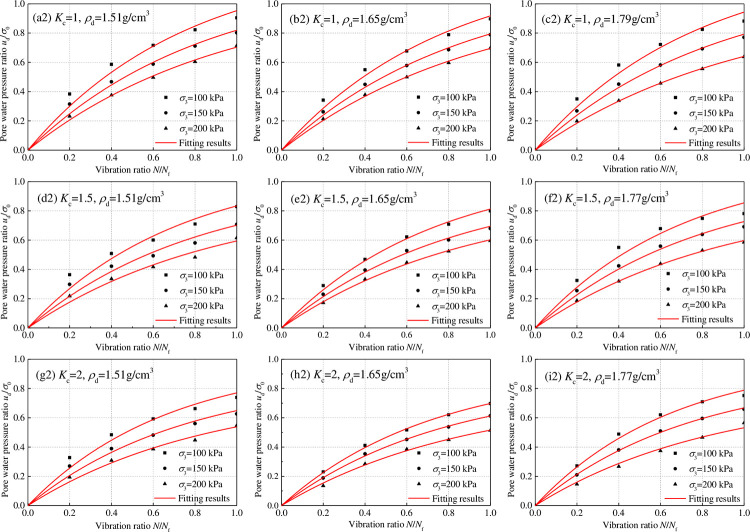
Relationship between dynamic pore water pressure ratio *u*_d_/*σ*_0_ and vibration ratio *N*/*N*_f_ of tailing silt.

The development law of dynamic pore water pressure is the variation law of dynamic pore water pressure with the vibration times in a test from the dynamic load application to the sample failure. The variation of dynamic pore water pressure is expressed by the dynamic pore water pressure ratio *u*_d_/*σ*_0,_. The vibration ratio is the ratio of the vibration time to the failure vibration time, expressed as *N*/*N*_f_. *u*_d_ is the dynamic pore water pressure (MPa), *σ*_0_ is the initial effective consolidation stress (*σ*_0_ = (*σ*_1_+*σ*_3_)/2), *N* is the vibration time, and *N*_f_ is the vibration time during liquefaction failure. The relation curve of the dynamic pore water pressure ratio *u*_d_/*σ*_0_ and vibration ratio of tailing silty sand and tailing silt under isotropic and anisotropic consolidation was obtained by analyzing the consolidated undrained dynamic triaxial test, as shown in Figs 7 and 8. Under dynamic load, the dynamic pore water pressure ratio *u*_d_/*σ*_0_ increased gradually with an increase in the vibration frequency ratio *N*/*N*_f_, increasing sample deformation. When the consolidation ratio *K*_c_ = 1.0, the last stage of the increase in dynamic pore water pressure ratio was abrupt, and the critical dynamic pore water pressure was close to the confining pressure. When the consolidation ratio *K*_c_ > 1.0, the development of the dynamic pore water pressure ratio gradually showed a downward trend, and the mutation in the last stage disappeared. The critical dynamic pore water pressure was less than the confining pressure; the larger the consolidation ratio, the smaller the critical dynamic pore water pressure.

The following were observed after further analysis of the influence of confining pressure, consolidation ratio, and dry density on the dynamic pore water pressure growth curve for the same tailings material: (1) Under the same consolidation ratio and dry density, the dynamic pore water pressure ratios decreased with an increase in confining pressures. The reduction ranged 0–10%; (2) Under the same confining pressure and consolidation ratio, the consolidation ratio significantly affected the development law of dynamic pore water pressure. The variation range was less than 5%; (3) Under the same confining pressure and dry density, the influence of the consolidation ratio on the development law of dynamic pore water pressure was significant. The dynamic pore water pressure ratio decreased with an increase in the consolidation ratio. The reduction ranged 0–30%. Therefore, the confining pressure and dry density do not influence the dynamic pore water pressure growth curve. By contrast, the consolidation ratio significantly influences the dynamic pore water pressure growth curve.

### 4.2 Typical increase curve of dynamic pore water pressure

**Fig 9:**
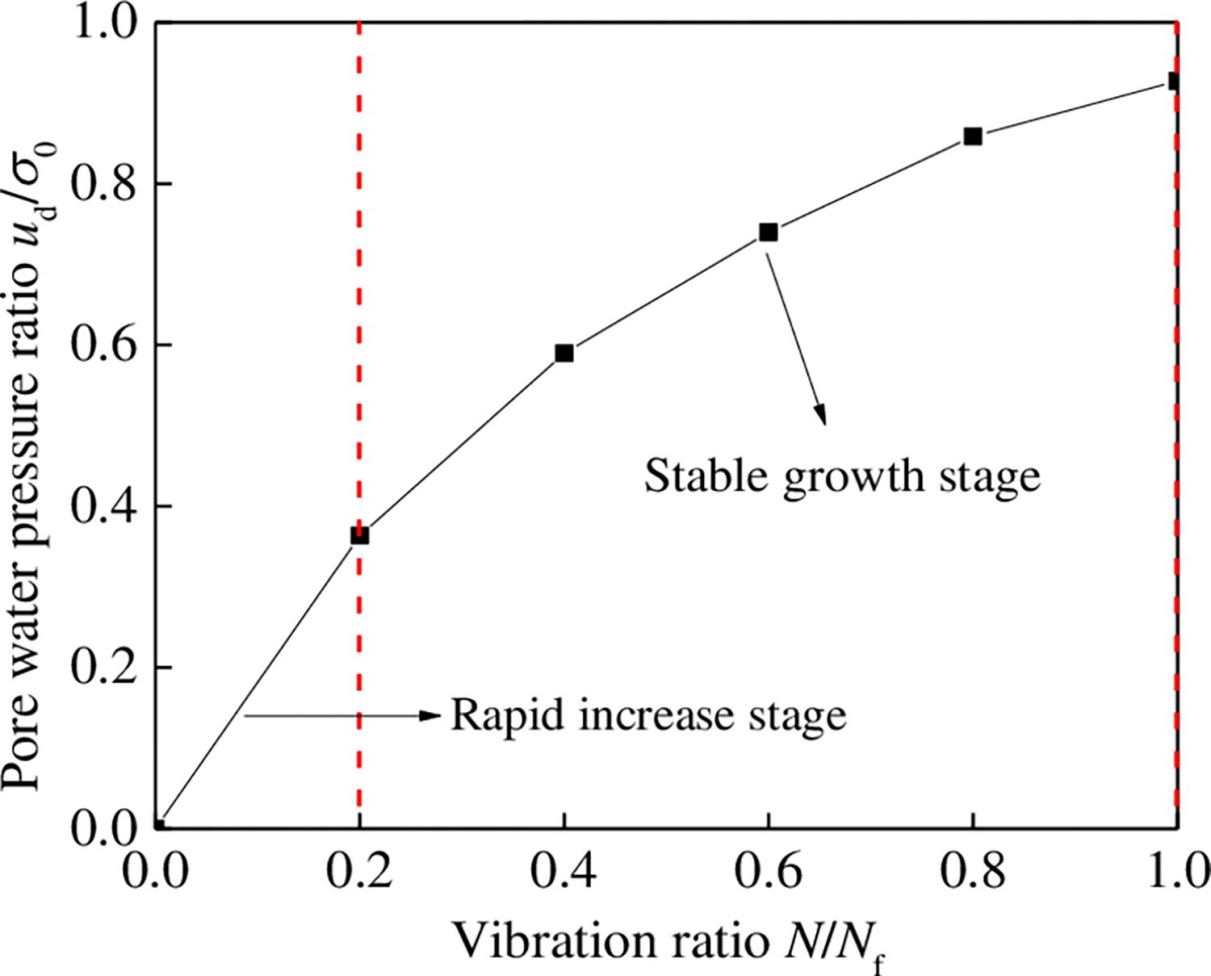
Typical curve of dynamic pore water pressure growth of tailing.

For different tailings materials, the dynamic pore water pressure of tailing silty sand was greater than that of tailing silt under the same conditions; that is, the finer the tailing particles, the smaller the dynamic pore water pressure ratio. However, the overall trend of dynamic pore water pressure growth curves is consistent. Fig 9 shows the typical dynamic pore water pressure growth curve of tailing silty sand and tailing silt during vibration under normalized conditions. According to the characteristics of the curve, the liquefaction process of the two tailings can be divided into two stages. The primary characteristics of each stage are as follows:

1. The stage of rapid increase in dynamic pore water pressure: At the initial stage of vibration, dynamic pore water pressure increases rapidly, and vibration ratio is in the range of 0–0.2. The dynamic pore water pressure ratio increases to approximately 0.3; the dynamic pore water pressure growth curve is roughly linear.
2. The stable growth stage of dynamic pore water pressure: When the vibration order ratio is greater than 0.2, the dynamic pore pressure ratio increases with an increase in the vibration order ratio until the sample is completely liquefied; the dynamic pore pressure growth curve is concave.

### 4.3 Variation law of critical dynamic pore water pressure during isotropic consolidation

When tailing sand is under isotropic consolidation (Figs 7A–7C and 8A–8C), the relationship between the critical dynamic pore water pressure of tailing silty sand and tailing silt under isotropic consolidation can be obtained according to Eq (2) and Table 2:

$$u_{\mathrm{cr}}=\sigma_{3}-2.4675\sigma_{d}$$ (5)

$$u_{\mathrm{cr}}=\sigma_{3}-2.6962\sigma_{d}+10.733$$ (6)

### 4.4 Variation law of critical dynamic pore water pressure during anisotropic consolidation

When tailing sand is under anisotropic consolidation (Figs 7D–7H and 8D–8H), the following conditions apply, according to Eq (3) and Table 2:

1. When the consolidation ratio *K*_c_ = 1.5, the dynamic effective internal friction angles of tailing silty sand and tailing silt are 20.3° and 17.6°, respectively, greater than the limit dynamic effective internal friction angle $\varphi_{d}^{c}=10.2{}^{\circ}$; thus, the critical dynamic pore water pressure ratio is λ > 0. The evolution law of critical dynamic pore water pressure under anisotropic consolidation is the same as that under isotropic consolidation. The critical dynamic pore water pressure of tailing silty sand and tailing silt is expressed as follows:
$$u_{\mathrm{cr}}=0.5294\sigma_{3}-0.9412\sigma_{d}$$ (7)
$$u_{\mathrm{cr}}=0.4232\sigma_{3}-1.1536\sigma_{d}+5.359.$$ (8)
2. When the consolidation ratio *K*_c_ = 2, the dynamic effective internal friction angles of tailing silty sand and tailing silt are 26.6° and 25.6°, respectively, greater than the limit dynamic effective internal friction angle $\varphi_{d}^{c}=19.5{}^{\circ}$; thus, the critical dynamic pore water pressure ratio is λ > 0. The critical dynamic pore water pressure of tailing silty sand and tailing silt is expressed as follows:
$$u_{\mathrm{cr}}=0.3833\sigma_{3}-0.167\sigma_{d}$$ (9)
$$u_{\mathrm{cr}}=0.3213\sigma_{3}-0.6787\sigma_{d}+3.6291.$$ (10)

Therefore, according to Eqs (5) and (6), the following apply under the condition of isotropic consolidation: For a tailing material, the critical dynamic pore water pressure is positively correlated with the confining pressure and negatively correlated with the dynamic stress amplitude. For different tailing materials, under the same confining pressure and dynamic stress amplitude, the critical dynamic pore water pressure is positively correlated with the average particle size of tailings. From Eq (7)–(10), under the anisotropic consolidation condition, the critical dynamic pore water pressure ratios of tailing silty sand and tailing silt are greater than 0, and the evolution law of critical dynamic pore water pressure is the same as that under isotropic consolidation. Therefore, the theoretical explanation of the influencing factors of dynamic pore water pressure evolution of tailings during isotropic consolidation proposed in the first section of the article is verified.

## 5 Dynamic pore water pressure growth model of tailings

### 5.1 Isotropic consolidation

Figs 7(A)–7(C) and 8(A)–8(C) show the relationship between the dynamic pore water pressure ratio *u*_d_/*σ*_0_ and vibration ratio *N*/*N*_f_ of tailing silty sand and tailing silt under isotropic consolidation (*K*_c_ = 1). Under isotropic consolidation, relatively small dynamic load could easily cause liquefaction failure of saturated sand. According to $u_{d}=\sigma_{0}=(\sigma_{1}+\sigma_{3})/2=\sigma_{3}$, when liquefaction occurs, the dynamic pore water pressure reaches the confining pressure and is close to the initial effective consolidation stress; thus, the dynamic pore water pressure ratio *u*_d_/*σ*_0_ is approximately equal to 1. Under this consolidation condition, the dynamic pore water pressure ratio *u*_d_/*σ*_0_ and vibration ratio *N*/*N*_f_ change from 0 to 1, and the regularity is evident. The most studied dynamic pore water pressure model of saturated sand is isotropic consolidation.

Seed et al. [5] performed undrained dynamic triaxial tests on saturated sand under isotropic consolidation. According to the test results, a dynamic pore water pressure stress model for general saturated sand under isotropic consolidation was proposed, and its applicability was widely recognized.

$$\frac{u_{d}}{\sigma_{0}}=\frac{2}{\pi }\arcsin{\left(\frac{N}{N_{f}}\right)}^{1/\theta },$$ (11)

where *θ* is the test constant (*θ =* 0.7).

The above equation is proposed for general saturated sand, which is inappropriate for tailing. Dynamic pore water pressure models for tailings have also been proposed in several studies, as shown in Table 4.

**Table 4:** Dynamic pore water pressure correction model proposed by predecessors.

Research object	Specific Equation	Test parameter value	Scholars
Copper tailings	$\frac{u_{d}}{\sigma_{0}}=\frac{4}{\pi }\arctan{\left(\frac{N}{N_{f}}\right)}^{1/2\theta }$	*θ* = 2.0	Zhang et al. [28]
Copper tailings silty sand	$\frac{u_{d}}{\sigma_{0}}=\frac{a}{\pi }\arctan{\left(\frac{1.5N}{N_{f}}\right)}^{1/2\theta }$	*a* = 0.94, *θ* = 1.3	Liu et al. [29]
Iron ore tailings silty sand	$\frac{u_{d}}{\sigma_{0}}=\frac{a}{\pi }\tan{\left(4\frac{N}{N_{f}}\right)}^{1/2\theta }$	*a* = 0.25, *b* = 1.735	Wang et al. [36]

**Fig 10:**
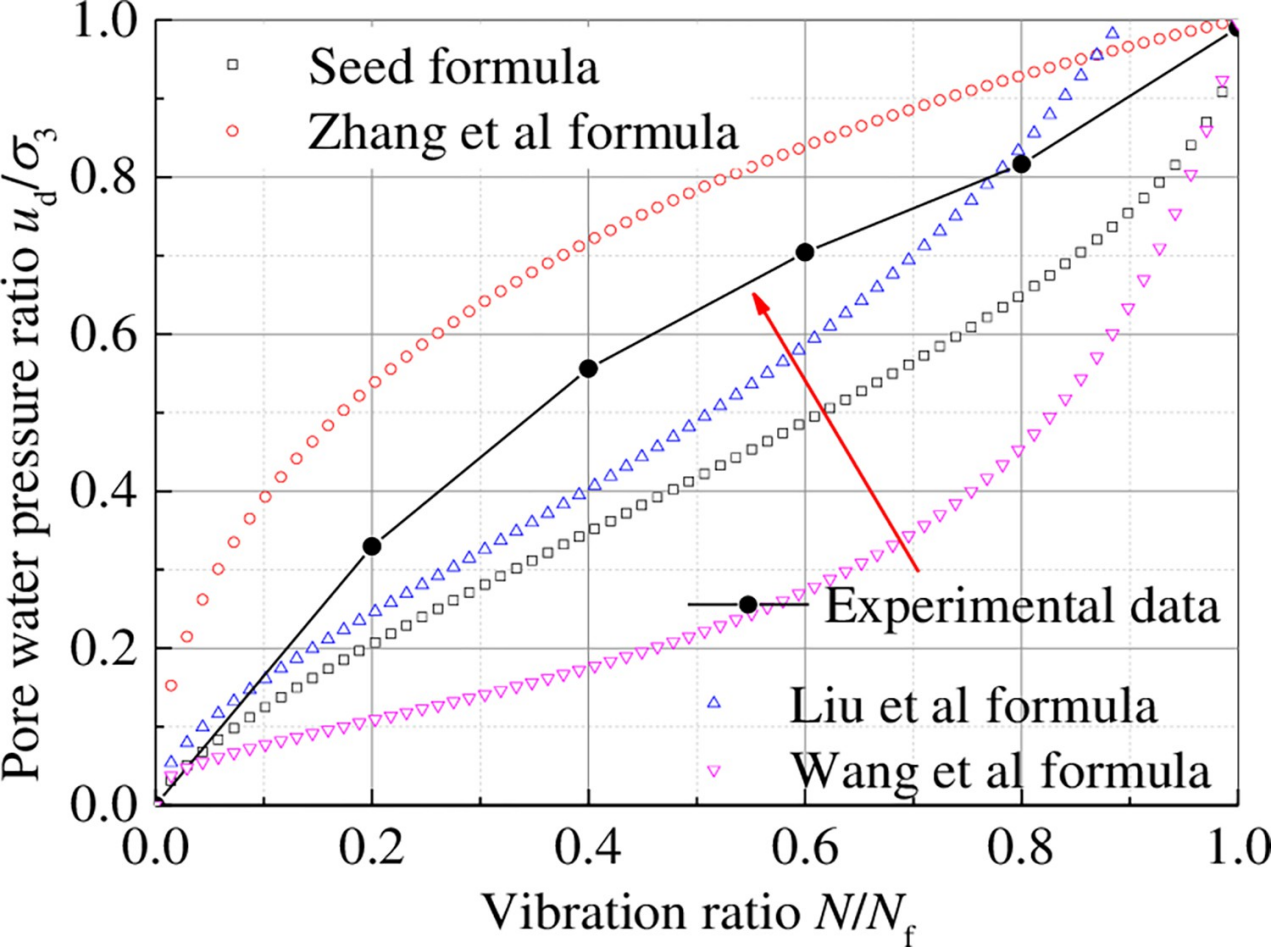
Relation curve between dynamic pore water pressure ratio and vibration ratio of common dynamic pore water pressure stress model.

These dynamic pore water pressure stress models and test data were plotted into a diagram, as shown in Fig 10. The dynamic pore water pressure growth process of tailings materials explored in this study differs under dynamic load from that of general sand materials. The primary difference is that the dynamic pore water pressure of general saturated sand increases steadily from beginning to failure, whereas the dynamic pore water pressure growth process of tailings materials in the middle of vibration is relatively slow. When it reaches the critical state, the dynamic pore water pressure of the sample rises rapidly until liquefaction failure. The equation proposed in previous studies cannot clearly define the dynamic pore water pressure model under different confining pressure. Therefore, the dynamic pore water pressure growth model of tailings materials under different conditions should be modified and explored further.

**Fig 11:**
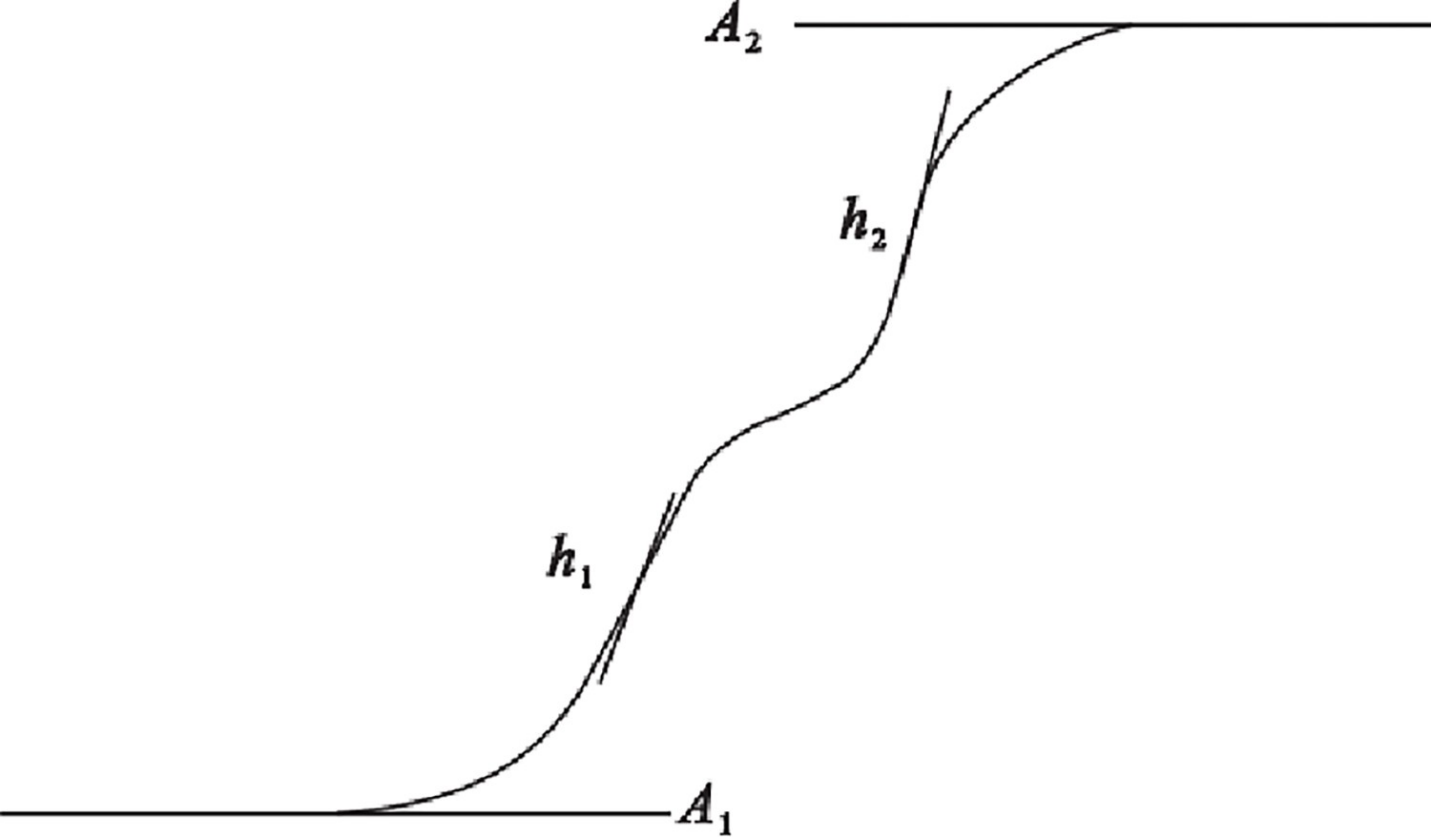
Schematic diagram of BiDoseResp function.

The above models are only applicable to isotropic consolidation but not to anisotropic consolidation. According to the test results, the characteristics of dynamic pore water pressure of tailings at each stage are consistent with the curve of the h1-A2 section in BiDoseResp function (Fig 11). Therefore, an exponential function model suitable for dynamic pore water pressure growth of tailing under isotropic consolidation was proposed, expressed as

$$\frac{u_{d}}{\sigma_{0}}=a(1-\exp(-a\frac{N}{N_{f}})),$$ (12)

where *a* is the test constant.

### 5.2 Anisotropic consolidation

Figs 7(D)–7(H) and 8(D)–8(H) show the relationship between the dynamic pore water pressure ratio *u*_d_/*σ*_0_ and vibration ratio *N*/*N*_f_ of tailing silty sand and tailing silt under anisotropic consolidation (*K*_c_ = 1.5 and *K*_c_ = 2). For anisotropic consolidation (*K*_c_ = *σ*_1_/σ_3_ > 1), owing to the large axial stress, greater dynamic load is required to cause liquefaction failure of saturated sand. Compared with isotropic consolidation, the law of dynamic pore water pressure rise during anisotropic consolidation is the same as that during isotropic consolidation. For anisotropic consolidation, owing to $u_{d}=\sigma_{0}=(\sigma_{1}+\sigma_{3})/2=(K_{c}+1)\sigma_{3}/2$, so $u_{d}=\sigma_{3}=2\sigma_{0}/(K_{c}+1)$ during liquefaction failure, the dynamic pore water pressure ratios vary from 0 to 2/(*K*_*c*_+1). Because 2/(*K*_*c*_+1)<1, the rising law of dynamic pore water pressure will be slower than that in isotropic consolidation.

The correction coefficient was added to Eq (12), and the expression of dynamic pore water pressure model under anisotropic consolidation was proposed, expressed as

$$\frac{u_{d}}{\sigma_{0}}=\xi \cdot a(1-\exp(-a\frac{N}{N_{f}})),$$ (13)

where *ξ* is the correction coefficient.

Through the comparative analysis of 27 groups of dynamic pore water pressure growth index model fitting test constants of tailing silty sand and tailing silt, we determined that *ξ* = 2/(*K*_c_+1). The dynamic pore water pressure correction model under different consolidation ratio can be obtained according to Eq (13):

$$\frac{u_{d}}{\sigma_{0}}=\frac{2}{K_{c}+1}a(1-\exp(-a\frac{N}{N_{f}}))$$ (14)

When *K*_c_ = 1, Eq (14) is equivalent to the dynamic pore water pressure correction model under isotropic consolidation of Eq (12).

Therefore, the dynamic pore water pressure model of a certain saturated tailing sand material can be uniquely determined. Eq (14) is the only model for dynamic pore water pressure correction of tailing sand material. For anisotropic consolidation, only the coefficient related to the consolidation ratio should be corrected. For isotropic consolidation, the coefficient is 1. Combined with the above conclusions, the rationality of the dynamic pore water pressure model with consolidation ratio *K*_c_ = 1.5 and 2 were verified.

When *K*_c_ = 1.5, the expression of the dynamic pore water pressure model is obtained according to Eq (14):

$$\frac{u_{d}}{\sigma_{0}}=\frac{4}{5}a(1-\exp(-a\frac{N}{N_{f}}))$$ (15)

When *K*_c_ = 2, the expression of the dynamic pore water pressure model is obtained according to Eq (14):

$$\frac{u_{d}}{\sigma_{0}}=\frac{2}{3}a(1-\exp(-a\frac{N}{N_{f}}))$$ (16)

### 5.3 Parameter analysis of dynamic pore water pressure model

**Fig 12:**
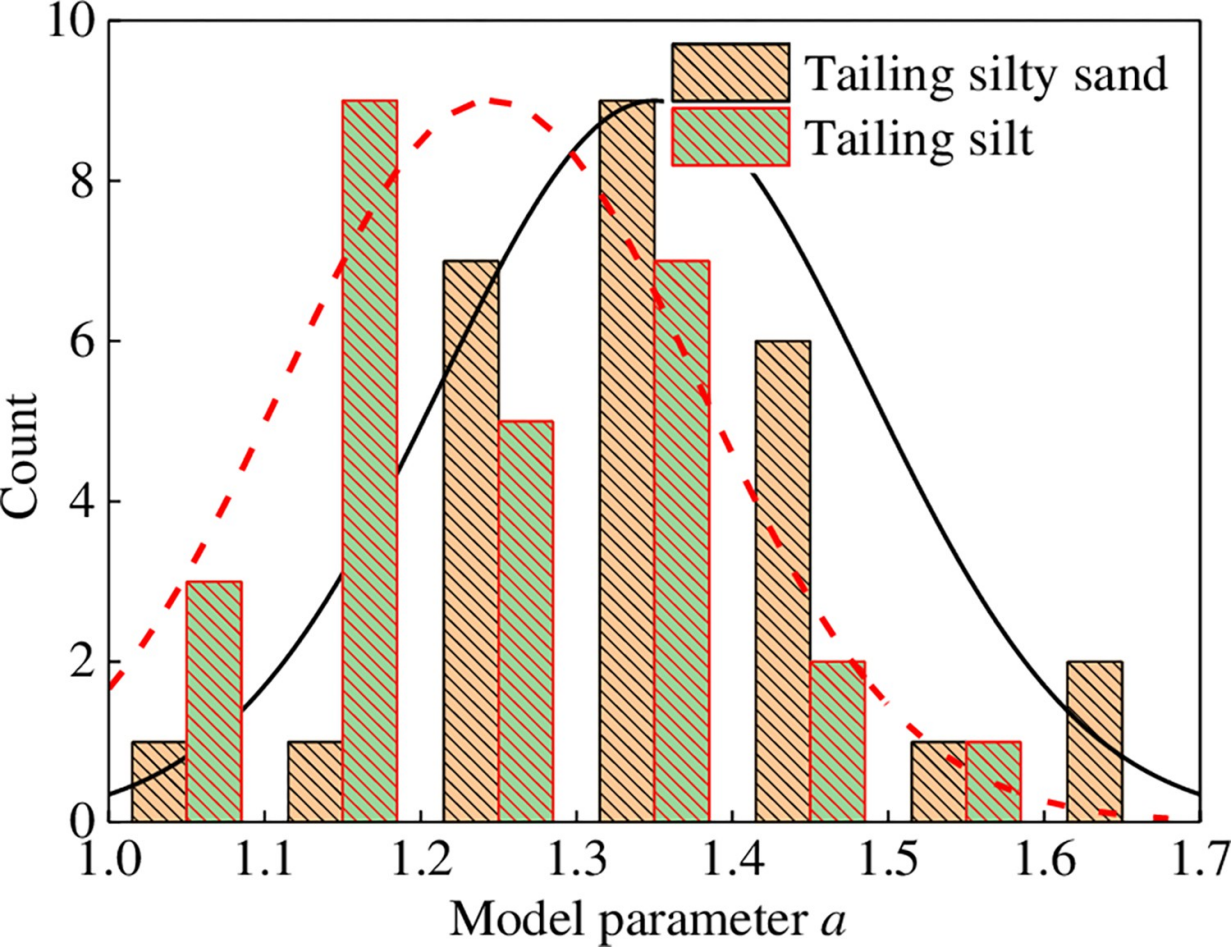
Normal distribution of model parameter *a*.

The dynamic pore water pressure growth exponential function model of Eq (14) was used to calculate the test curve of dynamic pore water pressure ratio *u*_d_/*σ*_0_ and vibration ratio *N*/*N*_f_ of tailing silty sand and tailing silt under isotropic and anisotropic consolidation; Table 5 shows the model fitting parameters. Figs 7 and 8 show that the fitting degree of the model to the test data is significantly high, and the correlation coefficients are more than 99%. According to Table 5, the fitting parameter *a* of the dynamic pore pressure growth model of tailing silty sand and tailing silt under different conditions is analyzed, as shown in Fig 12. The change rule of *a* under different conditions conforms to the normal distribution, and *a* varies from 1.0 to 1.6. The following were observed by analyzing the influence of confining pressure, consolidation ratio, and dry density on the fitting parameter *a* of the dynamic pore water pressure model for the same tailings material: (1) Under the condition of the same consolidation ratio and dry density, *a* decreases with an increase in confining pressure, with a decrease range of 0–15%; (2) Under the same confining pressure and consolidation ratio, *a* first decreases and then increases with an increase in dry density, and the variation range is within 8%; (3) Under the same confining pressure and dry density, the influence of consolidation ratio on the development law of dynamic pore water pressure is more significant. Moreover, *a* increases with an increase in consolidation ratio, with an increase range of 0–20%. For different tailings materials, the dynamic pore water pressure model fitting parameter *a* of tailing silty sand is slightly larger than that of tailing silt under the same conditions; that is, the finer the tailings particles, the smaller the *a*.

**Table 5:** Fitting parameters of dynamic pore water pressure growth model.

Tailings	*K*_c_	*ρ*_d_/(g/cm^3^)	σ_3_ = 100 kPa		σ_3_ = 150 kPa		σ_3_ = 200 kPa	
			*a*	*R* ^*2*^	*a*	*R* ^*2*^	*a*	*R* ^*2*^
Tailing silty sand	1	1.58	1.3884	0.981	1.3156	0.995	1.2116	0.986
		1.68	1.303	0.984	1.2088	0.981	1.0779	0.984
		1.79	1.328	0.988	1.2238	0.999	1.1136	0.997
	1.5	1.58	1.4972	0.999	1.3765	0.999	1.2642	0.991
		1.68	1.4407	0.997	1.3139	0.986	1.2258	0.983
		1.79	1.4798	0.997	1.3523	0.999	1.2205	0.991
	2	1.58	1.601	0.996	1.4624	0.997	1.318	0.997
		1.68	1.5688	0.996	1.4388	0.988	1.2869	0.976
		1.79	1.5992	0.999	1.4532	0.995	1.2976	0.978
Tailing silt	1	1.51	1.3071	0.977	1.1821	0.987	1.0709	0.997
		1.65	1.2741	0.989	1.1582	0.997	1.0625	0.999
		1.77	1.3098	0.974	1.1619	0.995	1.0101	0.999
	1.5	1.51	1.3998	0.982	1.2464	0.984	1.1121	0.998
		1.65	1.3645	0.997	1.2282	0.997	1.1183	0.999
		1.77	1.4125	0.972	1.2655	0.991	1.1121	0.999
	2	1.51	1.4913	0.977	1.3257	0.978	1.1708	0.989
		1.65	1.3942	0.999	1.2757	0.999	1.1381	0.999
		1.77	1.5158	0.994	1.3512	0.999	1.1615	0.991

## 6 Conclusion

The criterion for tailings sand liquefaction is primarily considered from a macro perspective. The liquefaction phenomenon itself is a macroscopic reflection of the sudden change in the tailings sand structure. To explain the liquefaction phenomenon of tailings sand, it is necessary to study the relationship between the development law of dynamic pore pressure and the change in the tailing structure. This study proposed an expression of the critical dynamic pore water pressure of tailing sand under different consolidation conditions through the limit equilibrium theory. The dynamic pore water pressure evolution of tailing silty sand and tailing silt under different dry densities, consolidation ratios, and confining pressures was analyzed through the undrained consolidation dynamic triaxial tests. The development law and growth model of dynamic pore water pressure of tailing sand under isotropic and anisotropic consolidation was expounded theoretically and experimentally. The main conclusions are as follows:

1. Under the condition of isotropic consolidation, the critical dynamic pore water pressure is positively correlated with the confining pressure and average particle size of tailings. The greater the amplitude of dynamic stress, the smaller the critical dynamic pore water pressure and the easier the tailings material can be damaged. There is a limit dynamic effective internal friction angle of tailings under anisotropic consolidation condition. The evolution law of critical dynamic pore water pressure can be judged according to the *φ*_d_ and $\varphi_{d}^{c}$ values; when $\varphi_{d}=\varphi_{d}^{c}$, λ = 0, the critical dynamic pore water pressure is independent of the confining pressure; when $\varphi_{d}>\varphi_{d}^{c}$, λ > 0, the critical dynamic pore water pressure is a positively correlated with the confining pressure; and when $\varphi_{d}<\varphi_{d}^{c}$, λ < 0, the critical dynamic pore water pressure is negatively correlated with the confining pressure.
2. The consolidation ratio significantly affects the dynamic pore pressure growth curve, whereas the confining pressure and dry density do not. Under isotropic consolidation conditions, the last stage of the increase in dynamic pore water pressure ratio is abrupt, and the critical dynamic pore water pressure is close to the confining pressure. Under isotropic and anisotropic consolidation conditions, the development of dynamic pore water pressure ratio gradually shows a downward trend, and the mutation in the last stage disappears. The critical dynamic pore water pressure is less than the confining pressure. Moreover, the larger the consolidation ratio, the smaller the critical dynamic pore water pressure. Regardless of the consolidation type, the critical dynamic pore water pressure is positively correlated with the confining pressure and negatively correlated with the dynamic stress amplitude. For different tailings materials, the critical dynamic pore water pressure is positively correlated with the average particle size of the tailings.
3. The dynamic pore water pressure growth process of tailing silty sand and tailing silt can be divided into two stages: dynamic pore water pressure rapid and stable growths. The development law of the two types of tailings can be described by the dynamic pore water pressure growth exponential correction model under different consolidation conditions. The dynamic pore water pressure growth exponential model only needs to modify the coefficient related to the consolidation ratio under the isobaric and eccentric consolidation conditions. The dynamic pore water pressure model of a certain saturated tailing sand material can be uniquely determined.
